# The Presence of Seminal Plasma during Liquid Storage of Pig Spermatozoa at 17 °C Modulates Their Ability to Elicit In Vitro Capacitation and Trigger Acrosomal Exocytosis

**DOI:** 10.3390/ijms21124520

**Published:** 2020-06-25

**Authors:** Ana Paula Pinoti Pavaneli, Sandra Recuero, Bruna Resende Chaves, Estela Garcia-Bonavila, Marc Llavanera, Elisabeth Pinart, Sergi Bonet, André Furugen Cesar De Andrade, Marc Yeste

**Affiliations:** 1 Biotechnology of Animal and Human Reproduction (TechnoSperm), Institute of Food and Agricultural Technology, University of Girona, ES-17003 Girona, Spain; anapavaneli@usp.br (A.P.P.P.); sandra.recuero@udg.edu (S.R.); brunarufla@gmail.com (B.R.C.); estela.garcia@udg.edu (E.G.-B.); marc.llavanera@udg.edu (M.L.); elisabeth.pinart@udg.edu (E.P.); sergi.bonet@udg.edu (S.B.); 2 Unit of Cell Biology, Department of Biology, Faculty of Sciences, University of Girona, E-17003 Girona, Spain; 3 Laboratory of Andrology and Technology of Swine Embryos, Department of Animal Reproduction, School of Veterinary Medicine and Animal Science, University of São Paulo, BR-13635-900 Pirassununga, Brazil; andrefc@usp.br; 4 Department of Veterinary Medicine, Federal University of Lavras, BR-37200-000 Lavras, Brazil

**Keywords:** spermatozoa, seminal plasma, in vitro capacitation, acrosomal exocytosis

## Abstract

Although seminal plasma is essential to maintain sperm integrity and function, it is diluted/removed prior to liquid storage and cryopreservation in most mammalian species. This study sought to evaluate, using the pig as a model, whether storing semen in the presence of seminal plasma affects the sperm ability to elicit in vitro capacitation and acrosomal exocytosis. Upon collection, seminal plasma was separated from sperm samples, which were diluted in a commercial extender, added with seminal plasma (15% or 30%), and stored at 17 °C for 48 or 72 h. Sperm cells were subsequently exposed to capacitating medium for 4 h, and then added with progesterone to induce acrosomal exocytosis. Sperm motility, acrosome integrity, membrane lipid disorder, intracellular Ca^2+^ levels, mitochondrial activity, and tyrosine phosphorylation levels of glycogen synthase kinase-3 (GSK3)α/β were determined after 0, 2, and 4 h of incubation, and after 5, 30, and 60 min of progesterone addition. Results showed that storing sperm at 17 °C with 15% or 30% seminal plasma led to reduced percentages of viable spermatozoa exhibiting an exocytosed acrosome, mitochondrial membrane potential, intracellular Ca^2+^ levels stained by Fluo3, and tyrosine phosphorylation levels of GSK3α/β after in vitro capacitation and progesterone-induced acrosomal exocytosis. Therefore, the direct contact between spermatozoa and seminal plasma during liquid storage at 17 °C modulated their ability to elicit in vitro capacitation and undergo acrosomal exocytosis, via signal transduction pathways involving Ca^2+^ and Tyr phosphorylation of GSK3α/β. Further research is required to address whether such a modulating effect has any impact upon sperm fertilizing ability.

## 1. Introduction

Seminal plasma is composed of a mixture of secretions produced by the accessory glands of the male reproductive tract, and contains a variety of constituents, such as proteins, lipids, steroid hormones, and ions [1]. Seminal plasma also contains extracellular vesicles (exosomes and microvesicles) [2,3] that infiltrate into the sperm membrane, thereby maintaining plasma membrane integrity [4]. These vesicles, which are also involved in the immune-related gene regulation in the uterus [5], are known to contain a repertoire of mRNA and small non-coding RNAs (microRNAs, miRNA; piwi-interacting RNAs, piRNA) that have been related to spermatogenesis and embryogenesis [6,7,8]. The mixture of spermatozoa with seminal plasma upon ejaculation underpins the idea that it is essential for these cells. In effect, despite not being well understood, the role of seminal plasma as a modulator of sperm function has become increasingly apparent in the last years. For this reason, its regulatory function on specific sperm parameters, such as motility and membrane permeability/fluidity, makes its use attractive to assisted reproductive technology [9].

In the case of cryopreservation protocols, removing extender and seminal plasma is a required step to increase sperm concentration and cryotolerance [10]. Related to this and considering the loss of the natural protection to spermatozoa provided by seminal plasma, previous studies have demonstrated that the reestablishment of that contact through post-thaw addition of seminal plasma can be beneficial for frozen-thawed spermatozoa, as it increases their motility, survival, and fertilizing ability [11,12,13]. Moreover, while other studies have investigated whether the presence of seminal plasma during liquid storage (15–20 °C) affects pig sperm function and survival, their results seem to be less consistent. Indeed, whilst some authors reported that removal of seminal plasma prior to liquid storage is a harmful practice, as it decreases sperm motility, viability, and acrosome integrity [14,15], others found that its elimination might be beneficial for sperm survival and in vivo fertilizing ability [16].

During capacitation, the process through which sperm cells acquire their ability to fertilize an oocyte, there are a series of changes that affect sperm motility, plasma membrane and acrosome integrity, membrane lipid disorder, mitochondrial activity, and Ca^2+^ homeostasis, and involve the phosphorylation of certain proteins [17,18,19,20]. Although seminal plasma proteins have been reported to inhibit sperm capacitation [21], the controversial results about to which extent the presence of seminal plasma is important to maintain sperm function during liquid storage [16] make it necessary to elucidate whether this prolonged interaction can affect the ability of mammalian sperm to elicit in vitro capacitation and trigger acrosomal exocytosis. Furthermore, mounting evidence in other species, like the sheep, indicates that not only is the overall sperm motility affected during in vitro capacitation but also the proportions of motile sperm subpopulations [22,23]. Therefore, whether storing pig sperm at 17 °C in the presence of seminal plasma tweaks the dynamics of these sperm subpopulations during in vitro capacitation also warrants further research.

Against this background, the present study aimed to evaluate how the presence of seminal plasma during liquid storage at 17 °C influences the ability of pig sperm to elicit in vitro capacitation and trigger acrosomal exocytosis. With this purpose, seminal plasma was separated from sperm upon collection. Thereafter, sperm samples were diluted in a commercial extender, supplemented with 15% or 30% seminal plasma, and stored at 17 °C for 48 h or 72 h. Control samples did not contain seminal plasma. After liquid storage, sperm samples were exposed to capacitating medium for 4 h; at this time point, acrosomal exocytosis was induced through the addition of progesterone. The following parameters were evaluated: plasma membrane and acrosome integrity, membrane lipid disorder, mitochondrial membrane potential, intracellular Ca^2+^ levels, and sperm motility and motile sperm subpopulations. Furthermore, tyrosine phosphorylation of glycogen synthase kinase-3 (GSK3) isoforms (α, β) was measured through immunoblotting, as this protein has been shown to be involved in the control of sperm motility, capacitation, and acrosome reaction in the pig [24] and other species [25,26].

## 2. Results

### 2.1. Sperm Motility

#### 2.1.1. Total and Progressive Sperm Motility

**Figure 1:**
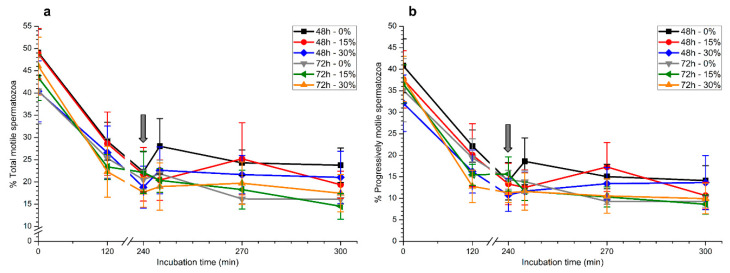
Percentages of total (**a**) and progressively motile spermatozoa (**b**) during in vitro capacitation and progesterone-induced acrosomal exocytosis (300 min) after previous storage of spermatozoa at 17 °C with different concentrations of seminal plasma (0%, 15%, and 30%) for 48 h or 72 h. Grey arrow indicates the time at which 10 μg/mL progesterone was added to induce acrosomal exocytosis (i.e., 240 min). No significant differences (*p* > 0.05) between treatments were observed. Data are shown as mean ± standard error of the mean (SEM) for seven independent experiments.

Although total and progressive sperm motility decreased significantly (*p* < 0.05) throughout incubation time, there was no effect (*p* > 0.05) of the presence of seminal plasma on any of these parameters when samples were stored at 17 °C for either 48 h or 72 h (Figure 1a,b).

#### 2.1.2. Motile Sperm Subpopulations

To determine the number of motile sperm subpopulations and evaluate their changes throughout in vitro capacitation and progesterone-induced acrosomal exocytosis, we first ran a principal component analysis (PCA) with all kinematic parameters; two extracted components that accounted for 80.20% of the total variance were identified (Table 1). 

The first principal component was mainly related to straight-line velocity, linearity, and straightness (VSL, straight-line velocity; VAP, average path velocity; LIN, linearity; WOB, wobble; STR, straightness; BCF, beat-cross frequency; and MAD, mean angular displacement), whereas the second one was highly related to curvilinear velocity and the amplitude of head displacement (VCL, curvilinear velocity; ALH, amplitude of lateral head displacement; and DANCE, VCL × ALH). After clustering spermatozoa utilizing the regression scores obtained in the PCA, we found three motile sperm subpopulations (Table 2). Spermatozoa belonging to subpopulations 1 and 2 (SP1 and SP2) were faster spermatozoa than those belonging to subpopulation 3 (SP3). In addition, whereas LIN, STR, and WOB were the highest in SP1, VCL and ALH reached their maximum in SP2. In contrast, VSL, VAP, and BCF values were similar between SP1 and SP2. The highest DANCE value was found in SP2, whereas the lowest MAD value was found in SP1. With the exception of MAD, all the other parameters were much lower in SP3. 

**Figure 2:**
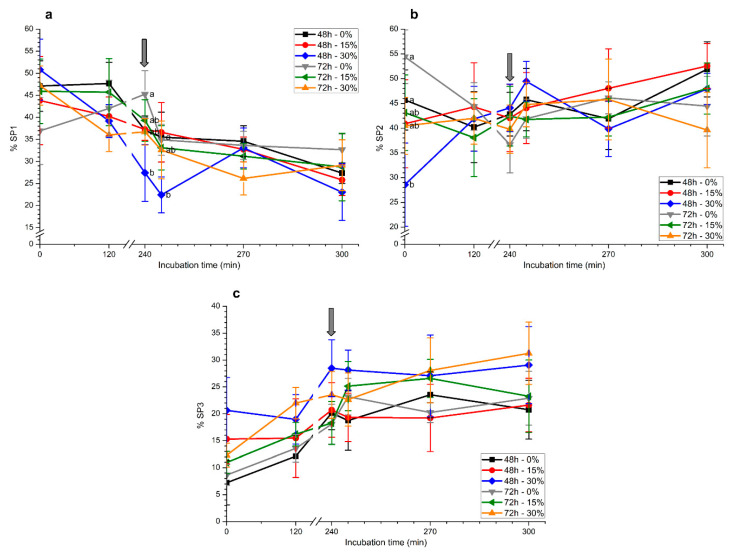
Percentages of motile sperm populations ((**a**) SP1, **(b**) SP2, and (**c**) SP3) during in vitro capacitation and progesterone-induced acrosomal exocytosis after previous storage of spermatozoa at 17 °C with different concentrations of seminal plasma (0%, 15%, and 30%) for 48 h or 72 h. Grey arrow indicates the time at which 10 μg/mL progesterone was added to induce acrosomal exocytosis (i.e., 240 min). Different letters mean significant (*p* < 0.05) differences between treatments at a given time point. Data are shown as mean ± SEM for seven independent experiments.

The aforementioned motile sperm subpopulations were monitored throughout in vitro capacitation and progesterone-induced acrosomal exocytosis (Figure 2). Sperm samples stored with 30% seminal plasma for 48 h showed a marked decrease in SP1 (Figure 2a). The addition of progesterone led to a significant (*p* < 0.05) reduction in SP1 (5 min), the lowest value being observed in those samples that had been stored with 30% seminal plasma for 48 h. No significant differences between treatments were observed after 30 and 60 min of progesterone addition. 

With regard to SP2, spermatozoa stored without seminal plasma for 72 h showed the highest value at 0 h (Figure 2b). In spite of this, no significant differences between treatments were observed at the other time points. Finally, proportions of spermatozoa belonging to SP3 did not differ between treatments, storage, or incubation times (Figure 2c). 

### 2.2. Plasma Membrane and Acrosome Integrity

**Figure 3:**
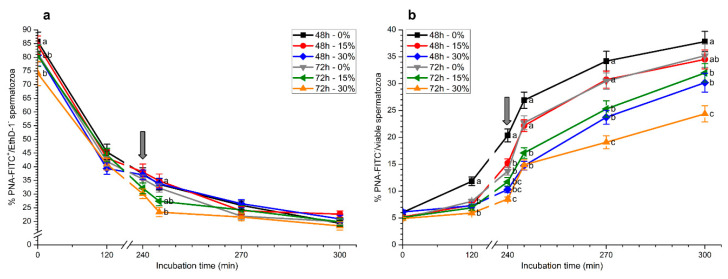
Percentages of viable spermatozoa with an intact acrosome (PNA-FITC^+^/EthD-1^−^; (**a**)) and with an exocytosed acrosome (PNA^−^) in relation to total viable spermatozoa (**b**) during in vitro capacitation and progesterone-induced acrosomal exocytosis (300 min) after previous storage of spermatozoa at 17 °C with different concentrations of seminal plasma (0%, 15%, and 30%) for 48 h or 72 h. Grey arrow indicates the time at which 10 μg/mL progesterone was added to induce acrosomal exocytosis (i.e., 240 min). Different letters mean significant (*p* < 0.05) differences between treatments at a given time point. Data are shown as mean ± SEM for seven independent experiments.

As expected, percentages of viable spermatozoa with an intact acrosome (PNA-FITC^+^/EthD-1^−^), which were significantly (*p* < 0.05) higher in samples stored for 48 h than in those stored for 72 h at the beginning of the experiment, decreased (*p* < 0.05) over the incubation period (Figure 3a). 

Whereas percentages of viable spermatozoa with an intact acrosome after 5 min of adding progesterone (245 min) were significantly (*p* < 0.05) lower in samples stored with 30% seminal plasma than in the other treatments, no significant differences (*p* > 0.05) in the other time points were observed (Figure 3a).

Percentages of viable spermatozoa exhibiting an exocytosed acrosome (PNA-FITC^−^/viable sperm) increased throughout incubation time and were significantly (*p* < 0.05) higher in samples stored without seminal plasma for 48 h than in the other treatments at 120 min and 240 min (Figure 3b). Previous storage with seminal plasma, especially in the treatment with 30% seminal plasma and 72 h storage, showed significantly (*p* < 0.05) lower percentages of viable spermatozoa exhibiting an exocytosed acrosome and a reduced response to the addition of progesterone (Figure 3b). 

### 2.3. Membrane Lipid Disorder

**Figure 4:**
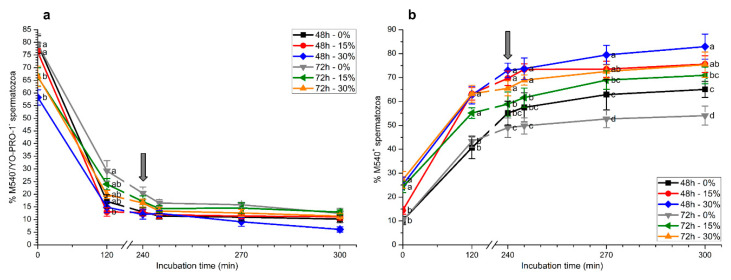
Percentages of viable spermatozoa with low membrane lipid disorder (M540^−^/YO-PRO-1^−^; (**a**)) and of M540^+^ spermatozoa (**b**) during in vitro capacitation and progesterone-induced acrosomal exocytosis (300 min) after previous storage of spermatozoa at 17 °C with different concentrations of seminal plasma (0%, 15%, and 30%) for 48 h or 72 h. Grey arrow indicates the time at which 10 μg/mL progesterone was added to induce acrosomal exocytosis (i.e., 240 min). Different letters mean significant (*p* < 0.05) differences between treatments at a given time point. Data are shown as mean ± SEM for seven independent experiments.

As expected, percentages of viable spermatozoa with low membrane lipid disorder (M540^−^/YO-PRO-1^−^) significantly (*p* < 0.05) decreased along incubation with capacitating medium (Figure 4a). At 120 min, percentages of viable spermatozoa with low membrane lipid disorder were significantly (*p* < 0.05) higher in samples stored without seminal plasma for 72 h than in those stored with 15% or 30% seminal plasma for 48 h. No significant differences were observed between treatments at the other incubation time times (240 min, 245 min, 270 min, and 300 min; Figure 4a). 

Percentages of spermatozoa with a positive M540 signal (M540^+^) showed a time-dependent increase (*p* < 0.05) during in vitro capacitation (Figure 4b). At 0 h, percentages of M540^+^ spermatozoa in samples that were stored with seminal plasma (15 or 30%) for 72 h and those stored with seminal plasma at 30% for 48 h were significantly (*p* < 0.05) higher than in those stored without seminal plasma. These significant differences (*p* < 0.05) were maintained throughout in vitro capacitation and after progesterone addition. At the end of the experiment, samples that were stored with seminal plasma at 30% for 48 h and 72 h showed significantly (*p* < 0.05) higher proportions of M540^+^ spermatozoa than those stored without seminal plasma. In addition, in the absence of seminal plasma, the percentages of M540^+^ spermatozoa were significantly higher (*p* < 0.05) in samples stored for 48 h than in those stored for 72 h (Figure 4b).

### 2.4. Intracellular Calcium Levels

**Figure 5:**
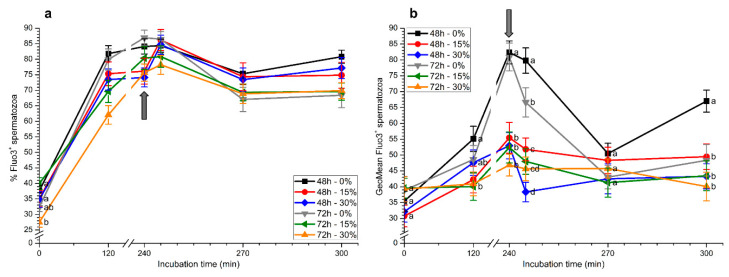
Percentages (**a**) and geometric mean intensity (**b**) of Fluo3^+^ spermatozoa during in vitro capacitation and progesterone-induced acrosomal exocytosis (300 min) after previous storage of spermatozoa at 17 °C with different concentrations of seminal plasma (0%, 15%, and 30%) for 48 h or 72 h. Grey arrow indicates the time at which 10 μg/mL progesterone was added to induce acrosomal exocytosis (i.e., 240 min). Different letters mean significant (*p* < 0.05) differences between treatments at a given time point. Data are shown as mean ± SEM for seven independent experiments.

Percentages of Fluo3^+^ spermatozoa (Fluo3^+^) increased progressively throughout incubation in capacitating medium (Figure 5a). At 2 h and 4 h, percentages of Fluo3^+^ spermatozoa were significantly (*p* < 0.05) higher in samples stored without seminal plasma, irrespective of the time of storage (48 h or 72 h). In addition, percentages of Fluo3^+^ spermatozoa at 2 h in samples stored with 30% seminal plasma for 72 h were significantly (*p* < 0.05) lower than in the other treatments. After 5 and 30 min of progesterone addition, no significant differences between treatments were observed. However, at the end of the experiment, percentages of Fluo3^+^ spermatozoa were significantly (*p* < 0.05) higher in samples stored without seminal plasma for 48 h than in those stored with or without seminal plasma for 72 h.

With regard to Fluo3^+^ intensity, maximum values of this parameter were observed after 4 h of incubation in samples stored for 48 h or 72 h in the absence of seminal plasma, in a similar fashion to that observed for the percentages of Fluo3^+^ sperm (Figure 5b). Subsequent to progesterone addition (5 min after IVAE), significantly (*p* < 0.05) higher values were found in samples stored without seminal plasma for 48 h and 72 h. In contrast, samples stored with 30% seminal plasma for 48 h and 72 h showed the lowest values of this parameter. Furthermore, whereas all sperm samples displayed similar values 30 min after progesterone addition, the Fluo3^+^ intensity was significantly (*p* < 0.05) higher in samples stored in the absence of seminal plasma for 48 h than in the other treatments (Figure 5b).

**Figure 6:**
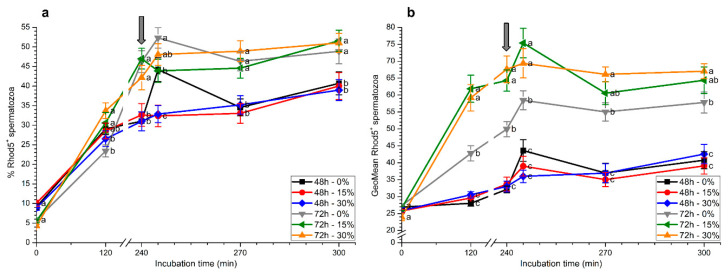
Percentages (**a**) and geometric mean intensity (**b**) of Rhod5^+^ spermatozoa during in vitro capacitation and progesterone-induced acrosomal exocytosis (300 min) after previous storage of spermatozoa at 17 °C with different concentrations of seminal plasma (0%, 15%, and 30%) for 48 h or 72 h. Grey arrow indicates the time at which 10 μg/mL progesterone was added to induce acrosomal exocytosis (i.e., 240 min). Different letters mean significant (*p* < 0.05) differences between treatments at a given time point. Data are shown as mean ± SEM for seven independent experiments.

Percentages of Rhod5^+^ spermatozoa increased progressively during in vitro capacitation and, after 2 h of incubation, they were significantly (*p* < 0.05) higher in samples stored in the presence of seminal plasma (15% or 30%) for 72 h than in the others (Figure 6a). At 4 h, percentages of Rhod5^+^ spermatozoa in samples stored for 72 h were significantly (*p* < 0.05) higher than those stored for 48 h, regardless of whether seminal plasma was present or absent. However, 5 min after progesterone addition, samples stored for 48 h without seminal plasma and those stored with and without seminal plasma for 72 h showed significantly (*p* < 0.05) higher percentages of Rhod5^+^ spermatozoa than those stored for 48 h with 15% and 30% seminal plasma. After 30 min and 60 min of progesterone addition, significantly (*p* < 0.05) higher percentages of Rhod5^+^ spermatozoa were observed in samples stored for 72 h than in those stored for 48 h, irrespective of the presence of seminal plasma (Figure 6a). 

Rhod5^+^ fluorescence intensity was significantly (*p* < 0.05) higher in samples stored for 72 h than in those stored for 48 h, with and without seminal plasma, throughout all the incubation period (Figure 6b). In the case of samples stored for 72 h, values observed when spermatozoa were kept with seminal plasma (15% or 30%) were significantly (*p* < 0.05) higher than when they were stored without this fluid. The subsequent addition of progesterone (5 min after progesterone addition) induced an increase in this parameter for all samples, and spermatozoa stored with seminal plasma for 72 h again showed significantly (*p* < 0.05) higher values for this parameter. At the end of the experiment, Rhod5^+^ fluorescence intensity was significantly (*p* < 0.05) higher in spermatozoa stored with 30% seminal plasma for 72 h than in the other treatments (Figure 6b).

### 2.5. Mitochondrial Membrane Potential

**Figure 7:**
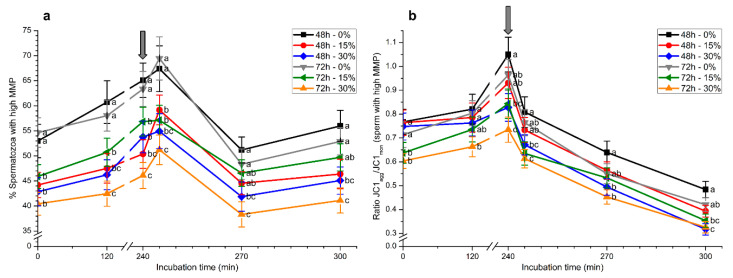
Percentages of spermatozoa with high mitochondrial membrane potential (MMP; (**a**)) and their JC1_agg_/JC1_mon_ ratios (**b**) during in vitro capacitation and progesterone-induced acrosomal exocytosis (300 min) after previous storage of spermatozoa at 17 °C with different concentrations of seminal plasma (0%, 15%, and 30%) for 48 h or 72 h. Grey arrow indicates the time at which 10 μg/mL progesterone was added to induce acrosomal exocytosis (i.e., 240 min). Different letters mean significant (*p* < 0.05) differences between treatments at a given time point. Data are shown as mean ± SEM for seven independent experiments.

Samples stored with 30% seminal plasma for 48 h or 72 h showed reduced percentages of spermatozoa with high mitochondrial membrane potential (MMP) at 0 h (*p* < 0.05; Figure 7a). These differences were maintained throughout the entirety of the experimental period. Samples stored without seminal plasma for 72 h showed significantly (*p* < 0.05) higher percentages of spermatozoa with high MMP than those stored with 15% or 30% seminal plasma. After 5 min of progesterone addition, samples stored without seminal plasma again showed significantly (*p* < 0.05) higher percentages of spermatozoa with high MMP. At the end of the experiment, only samples stored with 15% or 30% seminal plasma for 48 h and those stored with 30% seminal plasma for 72 h exhibited significantly (*p* < 0.05) lower percentages of spermatozoa with high MMP than those stored without seminal plasma (Figure 7a).

Looking at the JC1_agg_/JC1_mon_ ratios in the sperm population with high MMP, significantly (*p* < 0.05) higher values were observed in samples stored without seminal plasma for 72 h compared to those stored with 30% seminal plasma for 48 h (Figure 7b). After 5 min of progesterone addition and until the end of the experiment, JC1_agg_/JC1_mon_ ratios in the sperm population with high MMP were significantly (*p* < 0.05) higher in samples stored without seminal plasma than in those stored with 30% seminal plasma, irrespective of the time of storage (48 h or 72 h).

### 2.6. Tyrosine Phosphorylation Levels of GSK3α/β

**Figure 8:**
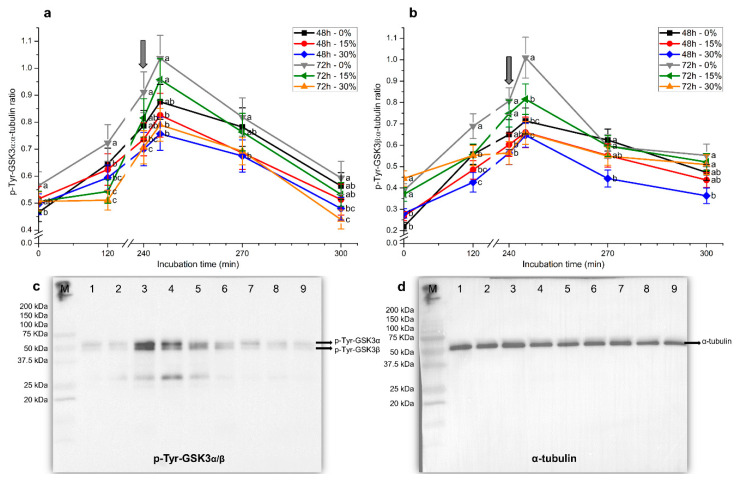
Relative tyrosine phosphorylation levels (using α-tubulin as a loading control) for glycogen synthase kinase-3 (GSK3)α (**a**) and GSK3β (**b**) during in vitro capacitation and progesterone-induced acrosomal exocytosis (300 min) after previous storage of spermatozoa at 17 °C with different concentrations of seminal plasma (0%, 15%, and 30%) for 48 h or 72 h. Grey arrow indicates the time at which 10 μg/mL progesterone was added to induce acrosomal exocytosis (i.e., 240 min). Results are shown as mean ± SEM for seven separate experiments. Different letters mean significant (*p* < 0.05) differences between treatments at a given time point. Representative blots for p-Tyr-GSK3α/β (**c**) and α-tubulin (**d**). Lanes: (M) protein ladder; (1) 72 h, 0% SP, 0 min; (2) 72 h, 0% SP, 120 min; (3) 72 h, 0% SP, 240 min; (4) 72 h, 0% SP, 245 min; (5) 72 h, 0% SP, 270 min; (6) 72 h, 0% SP, 300 min; (7) 72 h, 15% SP, 0 min; (8) 72 h, 15% SP, 120 min; (9) 72 h, 15% SP, 240 min.

**Figure 9:**
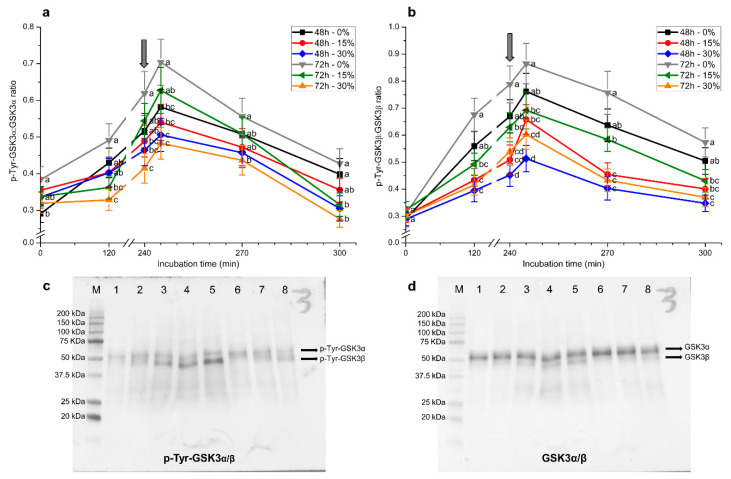
Relative tyrosine phosphorylation levels (using total GSK3α/β as a loading control) for GSK3α (**a**) and GSK3β (**b**) during in vitro capacitation and progesterone-induced acrosomal exocytosis (300 min) after previous storage of spermatozoa at 17 °C with different concentrations of seminal plasma (0%, 15%, and 30%) for 48 h or 72 h. Grey arrow indicates the time at which 10 μg/mL progesterone was added to induce acrosomal exocytosis (i.e., 240 min). Results are shown as mean ± SEM for seven separate experiments. Different letters mean significant (*p* < 0.05) differences between treatments at a given time point. Representative blots for p-Tyr-GSK3α/β (**c**) and α-tubulin (**d**). Lanes: (M) protein ladder; (1) 72 h, 0% SP, 0 min; (2) 72 h, 0% SP, 120 min; (3) 72 h, 0% SP, 240 min; (4) 72 h, 0% SP, 245 min; (5) 72 h, 0% SP, 270 min; (6) 72 h, 0% SP, 300 min; (7) 72 h, 15% SP, 0 min; (8) 72 h, 15% SP, 120 min.

Similar results were obtained for tyrosine phosphorylation levels of GSK3α/β, regardless of whether blots were normalized against α-tubulin or total GSK3α/β (Figure 8 and Figure 9). In spite of this, blots obtained using the former loading control were cleaner and the signal stronger than those observed with the latter. 

In the absence of seminal plasma, tyrosine phosphorylation levels of GSK3α in spermatozoa at 0 h were significantly (*p* < 0.05) higher in samples stored for 72 h than in those stored for 48 h (Figure 8a and Figure 9a). Tyrosine phosphorylation levels of GSK3α from 2 h and until the end of the experimental period were significantly (*p* < 0.05) higher in samples stored without than in those stored with seminal plasma, irrespective of the time of storage. Whereas tyrosine phosphorylation levels of GSK3α augmented along incubation, reaching maximum values after 5 min of progesterone addition, the extent of that increase was lower when samples were stored for 48 h or 72 h in the presence of 30% seminal plasma. In addition, tyrosine phosphorylation levels of GSK3α were the highest in spermatozoa stored without plasma for 72 h. Remarkably, samples stored in the presence of 30% seminal plasma showed the lowest tyrosine phosphorylation levels of GSK3α, especially at 270 min and 300 min of incubation (Figure 8a and Figure 9a).

Samples stored for 72 h, with and without seminal plasma, had significantly (*p* < 0.05) higher tyrosine phosphorylation levels of GSK3β than those stored for 48 h. These differences, however, were observed in blots normalized against α-tubulin (Figure 8b), but not in those normalized against total GSK3α/β (Figure 9b). 

In a similar fashion to that observed for p-Tyr-GSK3α levels, incubation of spermatozoa with capacitating medium led to an increase in p-Tyr-GSK3β levels, regardless of whether blots were normalized against α-tubulin or total GSK3α/β. These levels reached a peak after 5 min of progesterone addition and were significantly (*p* < 0.05) higher in sperm stored with seminal plasma for 72 h than in the other treatments. Again, the higher the concentration of seminal plasma with which sperm were stored, the lower the level of p-Tyr-GSK3β (Figure 8b and Figure 9b). At the end of the experiment, samples stored with 30% seminal plasma for 48 h showed the lowest tyrosine phosphorylation levels of GSK3β.

## 3. Discussion

Despite the recent insights into the biochemistry of seminal plasma along with on the biological performance of its components, the mechanisms through which it modulates sperm function still remain largely unknown [1,2,3,4,5,6,7,8]. In this context, the current study aimed to investigate whether storing pig semen at 17 °C with seminal plasma affects the sperm ability to elicit in vitro capacitation and trigger acrosomal reaction, and which intracellular mechanisms could be involved. 

Our results showed that the presence of seminal plasma during liquid storage of pig semen reduced the percentage of viable spermatozoa with an exocytosed acrosome, so that the longer the time of storage (72 h) and the higher the seminal plasma concentration (30%), the lower the sperm response to the induction of acrosomal exocytosis by progesterone. In addition, this previous storage with seminal plasma also affected intracellular calcium levels and membrane lipid disorder. This effect was observed as soon as sperm were separated from this fluid and were incubated in a capacitating medium (i.e., 0 h). Related to this, it is worth remembering that seminal plasma proteins are known to be modulators of sperm capacitation [27]. Amongst these proteins, heparin-binding spermadhesins have been associated with the stabilization of sperm membrane by covering the cell surface, thereby preventing early capacitation and acrosomal exocytosis [21]. Therefore, our results suggest that, in addition to the inhibition of early membrane changes during cooling, proteins and other constituents present in the seminal plasma, such as extracellular vesicles [4], maintain sperm membrane integrity and affect the ability to elicit in vitro capacitation and trigger acrosomal exocytosis.

The current work also sought to elucidate the intracellular mechanisms that underlie the effects of the presence of seminal plasma during liquid storage of pig semen at 17 °C. It is widely known that Ca^2+^, which was evaluated in this study by two separate fluorochromes, is involved in capacitation, hyperactivation, and acrosomal reaction [18,28,29]. Related to this, we observed that the intracellular mechanisms that regulate Ca^2+^ stores were modulated by the presence of seminal plasma and the time of storage. However, the impact on these stores relied upon whether they were located in the head (which are mainly stained by Rhod5) or in the mid-piece (which are mainly stained by Fluo3) [20]. On the one hand, spermatozoa stored in the presence of seminal plasma accumulated less Ca^2+^ (stained by Fluo3) in the mid-piece throughout in vitro capacitation, which could be related to the lower mitochondrial membrane potential observed in these treatments [30,31]. Related to this, it is worth mentioning that previous studies have demonstrated that in vitro sperm capacitation is associated with an increase in mitochondrial membrane potential [19,32] and Ca^2+^ levels in the sperm mid-piece [20]. Therefore, one could suggest that storing pig semen at 17 °C in the presence of seminal plasma affects the sperm ability to elicit in vitro capacitation and trigger acrosomal exocytosis through modulating mitochondrial function and altering Ca^2+^ storage in this compartment.

With regard to Ca^2+^ storage in the sperm head, there was an increase in the percentage of Rhod5^+^ spermatozoa in samples stored for 72 h, with and without seminal plasma, which suggests that the permeability of sperm head membrane to Ca^2+^ increases with a longer period of storage. This would match with the reduced percentage of viable spermatozoa with an intact acrosome observed after 245 min of incubation in samples stored with 30% seminal plasma for 72 h. However, while the presence of seminal plasma during liquid storage led to an increase in the geometric mean intensity of Rhod5^+^, this did not appear to be related to a higher sperm ability to undergo acrosome reaction following progesterone addition [33]. Whilst further studies are required to elucidate the separate Ca^2+^ channels that are involved in the different response of mid-piece and head stores, our results suggest that, in agreement with previous studies [20], the dynamics of these two stores differ and are reliant upon the time of storage and the presence of seminal plasma.

Previous studies have shown that high mitochondrial membrane potential is correlated with sperm motility [34,35,36] and fertilizing ability [37]. Although no significant differences between the presence and absence of seminal plasma were found in total and progressive sperm motility, we observed that storing pig semen at 17 °C with seminal plasma affected the structure of sperm motile subpopulations. In effect, sperm stored without seminal plasma exhibited higher percentages of SP1 (progressively motile and fastest spermatozoa) during in vitro capacitation and shortly after progesterone addition. These findings could be related to the higher mitochondrial membrane potential and accumulation of Ca^2+^ in the mitochondria observed in the absence of seminal plasma. Moreover, although no differences between treatments were observed with regard to SP2 and SP3, the percentages of spermatozoa belonging to SP2 tended to increase over the incubation time in samples stored with 30% seminal plasma. This increase was concomitant with a decrease in the percentages of spermatozoa belonging to SP1. In this study, SP2 was considered, in agreement with previous studies [38,39], as the hyperactivated sperm subpopulation. Therefore, one could suggest that storage of spermatozoa at 17 °C with seminal plasma could increase their ability to hyperactivate and increase membrane lipid disorder. This hypothesis would match with the higher membrane lipid disorder (M540^+^) observed in samples stored with 30% seminal plasma for 48 h. Hyperactivation is an essential step for sperm fertilizing ability, characterized by highly asymmetrical waveforms and an increase in the amplitude of flagellar bends [28]. Although changes in sperm motility patterns are known to depend on intracellular Ca^2+^ levels [28,29,40,41,42], our results indicate that seminal plasma affects head and mid-piece Ca^2+^ stores in a different manner. This could be related to the relevance of mitochondrial regulation during in vitro capacitation, as well as to the changes in the sperm head membrane prior to acrosomal exocytosis. Remarkably, while previous storage with seminal plasma appeared to increase the percentages of hyperactivated spermatozoa, they did not augment those of viable spermatozoa with an exocytosed acrosome. These findings would match with those reported in rams, since spermatozoa from this species are able to elicit in vitro capacitation without hyperactivation and its seminal plasma proteins induce hyperactivation while maintaining sperm in a decapacitated state [23].

Kinases and phosphatases take part in the molecular pathways that regulate, among other cell events, sperm motility and capacitation [43]. A crucial kinase in spermatozoa is glycogen synthase kinase-3 (GSK3), present in two isoforms (GSK3α and GSK3β) and ubiquitously expressed in mammalian tissues [44]. This protein is known to be related with sperm motility [24,45,46], sperm capacitation [24], acrosomal reaction [25], and fertilizing ability [26]. Serine and tyrosine phosphorylation of GSK3α and GSK3β has been established as a crucial regulatory mechanism for this protein. While phosphorylation at Ser21 or Ser9 (for α or β isoforms, respectively) is associated with GSK3-inhibition [47,48], tyrosine phosphorylation at Tyr279 or Tyr216 (for α or β isoforms, respectively) appears to increase its kinase activity [49,50]. However, some authors have suggested that the latter relationship is not as strict as the former, as pharmacological inhibition of GSK3 activity is not always correlated with reduced tyrosine phosphorylation [51]. While most works aiming at addressing role of GSK3 isoforms in mammalian spermatozoa have used serine phosphorylation as a method to determine its kinase activity [24,26,46], the involvement of tyrosine phosphorylation has been less studied. Herein, we investigated, for the first time, the changes of tyrosine phosphorylation of GSK3α and GSK3β that occur during in vitro capacitation and progesterone-induced acrosomal exocytosis. We used two different loading controls to normalize the intensity of the p-Tyr-GSK3α/β antibody (α-tubulin and total GSK3α/β); even though the results from the two approaches considerably coincided, signals were weaker and blots less clean when total GSK3α/β was used as the loading control. However, the fact that these two approaches were taken and agreed make the results more robust. Remarkably, we found that there was an increase of tyrosine phosphorylation in the two GSK3 isoforms when sperm were incubated under in vitro capacitating conditions, and that this increment was higher when samples were previously stored for a longer period (i.e., 72 h) without seminal plasma.

On the other hand, we observed that the presence of seminal plasma during liquid storage appeared to mitigate the capacitation-induced increase in tyrosine phosphorylation levels of GSK3α/β, which could be related to the reduced sperm ability to elicit in vitro capacitation [24]. Furthermore, storing sperm without seminal plasma for 72 h also increased tyrosine phosphorylation of GSK3β after progesterone addition. Since inhibition of GSK3β activity is related to a reduced sperm ability to undergo the acrosomal exocytosis induced by progesterone [25], our data suggest that storing pig sperm at 17 °C in the presence of seminal plasma for a longer period drops the kinase activity of GSK3β through a reduced phosphorylation of tyrosine residues, which results in a decreased percentage of spermatozoa that undergo acrosomal exocytosis.

Another interesting finding of our study was the lack of relationship between tyrosine phosphorylation of GSK3α/β and sperm motility. Previous studies conducted in other species reported that inhibition of GSK3α and GSK3β is required for activating sperm motility [24,46]. In this way, considering that tyrosine phosphorylation is related to an increase in the kinase activity of this protein [49,50], one would have expected more apparent differences between storage times and the presence/absence of seminal plasma with regard to sperm motility. However, the differences in tyrosine phosphorylation levels of GSK3α and GSK3β observed under different storage conditions had no impact on sperm motility parameters. While these results are in contrast with Vijayaraghavan et al. [45] who, working in bull spermatozoa, showed a positive correlation between tyrosine phosphorylation of GSK3 and sperm motility, the fact that our data were retrieved under capacitating conditions might explain these differences.

## 4. Materials and Methods

### 4.1. Materials

All reagents were purchased from Sigma-Aldrich (St. Louis, MO, USA), Boehringer-Mannheim (Mannheim, Germany), Merck (Darmstadt, Germany), and Panreac (Barcelona, Spain). All fluorochromes were provided by Thermo Fisher Scientific (Molecular Probes, Eugene, OR, USA) and were diluted with dimethyl sulfoxide (Sigma-Aldrich). Antibodies were acquired from Merck Millipore (Darmstadt, Germany), Agilent Technologies (Santa Clara, CA, USA), MyBioSource (San Diego, CA, USA), Cell Signaling (Danvers, MA, USA), and Dako (Glostrup, Denmark).

### 4.2. Semen Samples

Semen samples from seven boars were collected through the gloved-hand method by the technical staff of a local farm (Semen Cardona, S.L., Cardona, Spain). Handling of boars was performed in accordance with the EU Directive 2010/63/EU for animal experiments and the Animal Welfare Law issued by the Regional Government of Catalonia (Generalitat de Catalunya, Spain). However, the authors of this study did not manipulate any animal, as all samples were directly provided by the farm. Therefore, no specific ethical approval was required.

Upon collection, sperm-rich fraction were centrifuged at 2400× *g* and 17 °C for 5 min. The resulting supernatants (seminal plasma) were collected and stored at −20 °C. Sperm pellets were resuspended in a commercial extender (Duragen; Magapor, Ejea de los Caballeros, Spain) at a final concentration of 3 × 10^7^ spermatozoa/mL and distributed into aliquots of 50 mL, which were cooled down to 17 °C. Seminal plasma and three sperm aliquots were transported to the laboratory within 2 h post-collection, at −20 °C and at 17 °C, respectively.

### 4.3. Treatments and Semen Storage Procedures

For each replicate, the three 50 mL sperm aliquots were pooled and split into six fractions. Seminal plasma was thawed and added as follows: (1) control treatment (25 mL diluted semen with no seminal plasma), (2) 15% seminal plasma (21.25 mL diluted semen + 3.75 mL seminal plasma), and (3) 30% seminal plasma (17.5 mL diluted semen + 7.5 mL seminal plasma). Sperm concentration was adjusted to 1 × 10^7^ spermatozoa/mL. Each treatment was prepared in duplicate for evaluation after 48 h and 72 h of storage at 17 °C.

### 4.4. In Vitro Capacitation and Progesterone-Induced Acrosomal Exocytosis

Following liquid storage at 17 °C for 48 h or 72 h, samples were centrifuged at 600× *g* and 17 °C for 10 min and the resulting pellets were resuspended in 25 mL of capacitating medium (CM). This medium was composed of 20 mM HEPES (20 mM 4-(2-hydroxyethyl)-1-piperazineethanesulfonic acid), 125 mM NaCl, 3.1 mM KCl, 5 mM glucose, 0.3 mM Na_2_HPO_4_, 0.4 mM MgSO_4_·7H_2_O, 4.5 mM CaCl_2_, 15 mM NaHCO_3_, 21.7 mM sodium l-lactate, 1 mM sodium pyruvate, and 5 mg/mL of bovine serum albumin (BSA); the pH was adjusted to 7.4. Spermatozoa were incubated at 38.5 °C and 5% CO_2_ for 4 h (Heracell 150; Heraeus Instruments GmbH, Osterode, Germany), as described by Ramió-Lluch et al. [32]. In vitro capacitated spermatozoa were evaluated at 0, 2, and 4 h. After 4 h of incubation, progesterone (final concentration: 10 µg/mL) was added to induce acrosomal exocytosis [52,53]. Spermatozoa were further incubated under the same conditions and evaluated after 5, 30, and 60 min of progesterone addition. At each relevant time point, separate aliquots were taken for motility and flow cytometry analyses, and for protein extraction. In the latter case, aliquots were centrifuged at 2400× *g* and 17 °C for 5 min and supernatants were discarded. Pellets were stored at −80 °C until protein extraction.

### 4.5. Computer-Assisted Sperm Analysis

Sperm motility was evaluated using a computer-assisted sperm analysis (CASA; Integrated Sperm Analysis System, ISAS, V1.0; Proiser, Valencia, Spain). Briefly, samples were warmed at 38 °C for 15 min and a droplet of 5 μL was placed onto a previously warmed (38 °C) Makler chamber (Sefi Medical Instruments, Haifa, Isarel). A minimum of 200 spermatozoa were analyzed per sample and three separate fields were taken; two technical replicates were evaluated. This CASA system is based upon the analysis of 25 consecutive, digitalized photographic images (each image is captured every 40 ms) per field at a magnification of 100× (negative phase-contrast field, Olympus BX41 microscope; Olympus Europe GmbH, Hamburg, Germany). In addition to the mean values of total (TMOT, %) and progressive motility (PMOT, %), the following individual kinematic parameters were evaluated: straight linear velocity (VSL, μm/s), which represents the average velocity measured in a straight line from the beginning to the end of a given sperm track; curvilinear velocity (VCL, μm/s), which is the average velocity measured over the actual point-to-point track followed by the cell; average path velocity (VAP, μm/s), which corresponds to the average velocity of the smoothest cell pathway; linearity (LIN, %), which is provided by the quotient of VSL/VCL; straightness (STR, %), which results from dividing VSL by VAP; wobble coefficient (WOB, %), the ratio of VAP/VCL; amplitude of lateral head displacement (ALH, μm); beat cross frequency (BCF, Hz), which is the frequency at which the sperm cell head crosses the average pathway; the combination of the lateral and forward movement of the head (DANCE, μm^2^/s), which results from multiplying VCL with ALH; and the mean angular displacement (MAD, degrees) [23,54]. A sperm cell was defined as being motile when VAP ≥ 10 µm/s and progressively motile when STR ≥ 45%. 

Individual kinematic parameters were used to determine the number and characteristics of motile sperm subpopulations and to evaluate whether their structure changed in response to the presence of seminal plasma during liquid storage at 17 °C, and throughout in vitro capacitation and progesterone-induced acrosomal exocytosis.

### 4.6. Flow Cytometry

Plasma and acrosome integrity, membrane lipid disorder, intracellular calcium levels, and mitochondrial membrane potential were evaluated by flow cytometry. These analyses were conducted using a Cell Laboratory QuantaSC cytometer (Beckman Coulter, Fullerton, CA, USA), after excitation through an argon ion laser (488 nm) set at a power of 22 mW. Cell diameter/volume (i.e., electronic volume, EV) was measured using the Coulter principle for volume assessment, as this system has forward scatter (FSC) replaced by electronic volume (EV). Before spermatozoa were stained with fluorochromes, we adjusted concentration to 1 × 10^6^ spermatozoa/mL in a final volume of 0.5 mL [55]. Samples were assessed at a sheath flow rate of 4.17 µL/min with 10,000 cells being acquired per analysis. Three independent replicates were evaluated. To capture fluorochrome signals, we used three different optical filters: FL1 (green fluorescence)—Dichroic/Splitter, DRLP: 550 nm, BP filter: 525 nm, detection width 505–545 nm; FL2 (orange fluorescence)—DRLP: 600 nm, BP filter: 575 nm, detection width: 560–590 nm; FL3 (red fluorescence)—LP filter: 670 nm, detection width: 655–685 nm. Debris (particle diameter < 7 µm) and aggregates (particle diameter > 12 µm) were excluded from the analysis by gating the particles on the basis of EV/side scatter (SSC) plots. Data obtained from flow cytometry analyses were corrected following the protocol described by Petrunkina et al. [56].

#### 4.6.1. Acrosome Integrity

Acrosome integrity was evaluated through co-staining with the lectin from *Arachis hypogaea* (peanut agglutinin, PNA) conjugated with fluorescein isothiocyanate (FITC) and ethidium homodimer (3,8-diamino-5-ethyl-6-phenylphenanthridinium bromide; EthD-1), as described by Rocco et al. [57]. First, spermatozoa were incubated with EthD-1 (final concentration: 2.5 µg/mL) at 37.5 °C for 5 min in the dark. Following this, samples were centrifuged at 2000× *g* and 16 °C for 30 s and then resuspended with PBS supplemented with 4 mg/mL BSA. Thereafter, samples were centrifuged at 2000× *g* and 16 °C for 30 s, and subsequently fixed and permeabilized by adding 100 µL ice-cold methanol (100%) for 30 s. Methanol was removed by centrifugation at 2000× *g* and 16 °C for 30 s, and pellets were resuspended with 250 µL PBS. Finally, samples were stained with PNA-FITC (final concentration: 2.5 µM) at 25 °C for 15 min in the dark, washed twice with PBS at 2000× *g* for 30 s, and resuspended in PBS. 

When stained samples were evaluated with the flow cytometer, four sperm populations were identified [58]: (i) viable sperm with an intact acrosome (PNA-FITC^+^/EthD-1^−^), (ii) viable sperm with an exocytosed acrosome (PNA-FITC^−^/EthD-1^−^), (iii) non-viable sperm with an intact acrosome (PNA-FITC^+^/EthD-1^+^), and (iv) non-viable sperm with an exocytosed acrosome (PNA-FITC^−^/EthD-1^+^). Fluorescence of EthD-1 was detected through FL3, and PNA-FITC fluorescence through FL1. Results are expressed as the percentage of viable sperm with an exocytosed acrosome (PNA-FITC^−^) in relation to the total viable sperm population (EthD-1^−^).

#### 4.6.2. Membrane Lipid Disorder

Membrane lipid disorder was assessed using the co-staining protocol for merocyanine-540 (M540) and YO-PRO-1, as described by Harrison et al. [59]. Sperm samples collected at each time point were incubated with M540 (final concentration: 2.6 µM) and YO-PRO-1 (final concentration: 25 nM) at 38 °C in the dark for 10 min. Fluorescence emitted by M540 and YO-PRO-1 was detected through FL3 and FL1, respectively. Sperm cells stained with M540 corresponded to those exhibiting high membrane lipid disorder, whereas those stained with YO-PRO-1 indicated the occurrence of early changes in their membrane permeability. Four populations were identified: (i) spermatozoa with no changes in membrane permeability and low membrane lipid disorder (M540^−^/YO-PRO-1^−^), (ii) spermatozoa with no changes in membrane permeability and high membrane lipid disorder (M540^+^/YO-PRO-1^−^), (iii) spermatozoa with changes in membrane permeability and low membrane lipid disorder (M540^−^/YO-PRO-1^+^), and (iv) spermatozoa with changes in membrane permeability and high membrane lipid disorder (M540^+^/YO-PRO-1^+^).

#### 4.6.3. Intracellular Calcium Levels

Intracellular calcium levels were evaluated with two different markers (Fluo3 and Rhod5). Fluo3 staining was performed following the protocol described by Harrison et al. [60] and modified by Kadirvel et al. [61], whereas Rhod5 staining was performed following the protocol described by Yeste et al. [20]. Whereas Fluo3 has more affinity for the Ca^2+^ stored in the mid-piece, Rhod5 preferentially stains that stored in the sperm head [20].

For Fluo3, spermatozoa were incubated at 38 °C in the dark for 10 min with Fluo3-AM (final concentration: 1 µM) and PI (final concentration: 12 µM), which were detected through FL1 and FL3, respectively. A total of four sperm populations could be identified: (i) viable spermatozoa with low levels of intracellular calcium (Fluo3^−^/PI^−^), (ii) viable spermatozoa with high levels of intracellular calcium (Fluo3^+^/PI^−^), (iii) non-viable spermatozoa with low levels of intracellular calcium (Fluo3^−^/PI^+^), and (iv) non-viable spermatozoa with high levels of intracellular calcium (Fluo3^+^/PI^+^). Fluo3 spill over into the FL3 channel (2.45%) and PI spill-over into the FL1 channel (28.72%) were compensated, and the geometric mean of Fluo3 intensity was recorded for all sperm populations.

On the other hand, spermatozoa were incubated at 38 °C for 10 min in the dark with Rhod5-N (final concentration: 5 µM) and YO-PRO-1 (final concentration: 25 nM). Filters used to detect the fluorescence from Rhod5 and YO-PRO-1 were FL3 and FL1, respectively. A total of four sperm populations were identified: (i) viable spermatozoa with low levels of intracellular calcium (Rhod5^−^/YO-PRO-1^−^), (ii) viable spermatozoa with high levels of intracellular calcium (Rhod5^+^/YO-PRO-1^−^), (iii) non-viable spermatozoa with low levels of intracellular calcium (Rhod5^−^/YO-PRO-1^+^), and (iv) non-viable spermatozoa with high levels of intracellular calcium (Rhod5^+^/YO-PRO-1^+^). Fluorescence from Rhod5 was compensated into the FL1 channel (3.16%), and the geometric mean of Rhod5 intensity was recorded for all sperm populations.

#### 4.6.4. Mitochondrial Membrane Potential

Mitochondrial membrane potential (MMP) was determined using JC1 (5,5′,6,6′-tetrachloro-1,1′,3,3′-tetraethylbenzimidazolylcarbocyanine iodide) fluorochrome, following the protocol described by Garner and Johnson [62]. With this purpose, sperm samples were incubated with JC1 (final concentration: 0.3 µM) at 38 °C in the dark for 30 min, and two sperm populations were distinguished: (i) spermatozoa with high mitochondrial membrane potential, and (ii) spermatozoa with low mitochondrial membrane potential. When MMP was high, JC1 inside mitochondria formed orange aggregates that were detected through FL2. At low MMP, JC1 remained as monomers and emitted green fluorescence that was detected through FL1 [63]. Geometric intensities of JC1_mon_ (FL1) and JC1_agg_ (FL2) were recorded and the ratio between JC1_agg_ and JC1_mon_ (JC1_agg_/JC1_mon_) was also calculated for each sperm population. FL1 spill-over into the FL2 channel was compensated (51.70%).

### 4.7. Tyrosine Phosphorylation Levels of GSK3α/β

Sperm pellets stored at −80 °C were thawed and resuspended in 400 µL ice-cold lysis buffer and maintained at 4 °C for 30 min under constant agitation. The lysis buffer was made up of 2% SDS, 1% Triton-X-100, 8 M Urea, 2 mM dithiothreitol (DDT), 0.5% Tween 20, and 50 mM Tris-HCl; the pH was adjusted to 7.4. On the day of use, the lysis buffer was added with 1% commercial protease inhibitor cocktail (Sigma-Aldrich), 1% phenylmethanesulfonyl fluoride (PMSF), and 0.15% sodium orthovanadate. Following this, samples were homogenized by sonication (50% amplitude; 10 long-lasting pulses; Bandelin Sonopuls HD 2070; Bandelin Electronic GmbH and Co., Heinrichstrasse, Berlin), and then centrifuged at 10,000× *g* and 4 °C for 15 min. Supernatants were carefully collected and total protein was quantified in triplicate with a detergent-compatible protein assay (DC Protein Assay; BioRad, Hercules, CA, USA). Standard curves were made with different concentrations of BSA (Quick Start Bovine Serum Albumin Standard; Bio-Rad).

A total of 10 µg of total protein was mixed with 2× Laemmli sample buffer and incubated at 90 °C for 5 min. Protein samples were subsequently loaded onto gradient commercial SDS-PAGE gels (Mini-Protean TGX Stain-Free gels; percentage acrylamide in the separating gel: 8–16%), together with a molecular weight marker (Precision Plus Protein All Blue Standards, Bio-Rad). Each gel was run at 20 mA under an initial voltage of 80 V and final voltage of 120–150 V through an electrophoresis system (Mini-PROTEAN Tetra Cell, Bio-Rad). Thereafter, proteins bands were transferred onto polyvinylidene fluoride membranes (PVDF; Immobilon-P; Merck Millipore) through a transfer system (Mini-Trans Blot Cell; Bio-Rad) at 240 mA for 2 h. Membranes were subsequently incubated at 4 °C overnight, and under constant agitation with a blocking solution consisting of 5% (*v*/*v*) BSA diluted in Tris-buffered saline containing Tween 20 (1 × TBS-Tween20). Following this, membranes were incubated with a primary anti-phospho-GSK3α/β (Tyr279/Tyr216) (ref. 05-413; Merck Millipore) antibody diluted 1:5000 (*v*/*v*) in blocking solution, under agitation and at room temperature for 1 h. After we washed the membranes three times (5 min per wash) with a solution made up of 10 mM Tris, 150 mM NaCl, and 0.05% Tween20 (pH = 7.3), they were incubated with a secondary anti-mouse polyclonal antibody conjugated with horseradish peroxidase (HRP; ref. P0260; Agilent Technologies) diluted 1:10,000 (*v*/*v*) in blocking solution, at room temperature for 1 h under constant agitation. Membranes were washed 5 times (5 min per wash) in the case of membranes that were further incubated with an anti-α-tubulin antibody and 10 times in that of membranes that were further incubated with anti-GSK3α plus anti-GSK3β antibodies. Reactive bands were subsequently visualized using a chemilumimnescent substrate (Immobilon Western Detection Reagents; Merck Millipore). Two reactive bands with different molecular weights corresponding to α (≈51 kDa) and β (≈47 kDa) GSK3 isoforms [24] were identified. No blocking peptide was available for this primary antibody, and thus no peptide blocking assay could be run. However, this primary antibody was raised against the human GSK3, whose sequence homology with regard to the pig is, according to Protein BLAST (Basic Local Alignment Search Tool) database (National Center for Biotechnology Information, NCBI, Bethesda, MD, USA), 96% (GSK3α) and 99% (GSK3β). Images were acquired with Genesys software (Synoptics Limited, Cambridge, United Kingdom). 

Anti-α-tubulin (ref. MABT205, Merck Millipore), anti-GSK3α (ref. 9338; Cell Signaling), and anti-GSK3β (ref. MBS8207657; MyBioSource) antibodies were separately used as internal standards to normalize the intensity of p-Tyr-GSK3α/β-bands [64]. In brief, membranes were stripped using a buffer made up of 1.5% glycine, 0.1% SDS, and 1% Tween20, and the pH was adjusted to 2.2. Membranes were incubated with an anti-α-tubulin antibody diluted at 1:100,000 (*v*/*v*) or anti-GSK3α plus anti-GSK3β antibodies diluted at 1:5000 (*v*/*v*) in blocking solution and then washed three times (5 min per wash) in the case of anti-α-tubulin and five times in that of anti-GSK3α plus anti-GSK3β antibodies. Subsequently, membranes were incubated with a secondary anti-mouse polyclonal antibody conjugated with horseradish peroxidase (HRP; ref. P0260; Agilent Technologies) diluted at 1:150,000 (*v*/*v*) in the case of the anti-α-tubulin antibody and at 1:10,000 (*v*/*v*) in the case of anti-GSK3α/β antibodies. After washing membranes 5 times in the case of anti-α-tubulin antibody and 10 times in that of anti-GSK3α plus anti-GSK3β antibodies, we used a chemilumimnescent substrate (Immobilon Western Detection Reagents; Merck Millipore) to visualize the bands corresponding to α-tubulin or GSK3α plus GSK3β [62]. Images were taken with the Genesys software (Synoptics Limited, Cambridge, United Kingdom).

Quantification of protein bands in blot images was performed with Quantity One 1-D Analysis Software (Bio-Rad). Values were expressed as the total signal intensity corresponding to pixel intensity units (density, mm^2^) present inside the boundary of the band. In addition, a background signal was excluded and the lowest intensity of a pixel was considered as zero [65]. Ratios between each p-Tyr-GSK3-band (i.e., α or β) and α-tubulin, or GSK3α/GSK3β (total) bands were calculated per lane. Although signals were clearer and blots cleaner when α-tubulin was used as an internal standard, differences between treatments with regard to these ratios were quite similar.

### 4.8. Statistical Analyses

Data were analyzed with a statistical package (IBM SPSS 25.0 for Windows; IBM Corp.; Chicago, IL, USA). First of all, normality (Shapiro–Wilk test) and homogeneity of variances (Levene test) were checked. Following this, the effects of treatment (0%, 15%, and 30% seminal plasma) and storage time (48 and 72 h) on sperm motility parameters, acrosome integrity, membrane lipid disorder, mitochondrial membrane potential, intracellular Ca^2+^ levels, and tyrosine phosphorylation of GSK3α/β (normalized against α-tubulin and total GSK3α/β) during in vitro capacitation and progesterone-induced acrosomal exocytosis were tested with a linear mixed model followed by a Sidak test for pair-wise comparisons. Treatment (presence/absence of seminal plasma) and storage conditions were the inter-subject factors, and the incubation time during in vitro capacitation and progesterone-induced acrosomal exocytosis was the intra-subject factor. All sperm parameters were considered as dependent variables.

Sperm subpopulations were set according to the procedure described by Luna et al. [23] and Estrada et al. [66] with minor modifications. First, the individual CASA parameters obtained for each sperm cell (VSL, VCL, VAP, LIN, STR, WOB, ALH, BCF, MAD, and DANCE) were used as independent variables in a principal component analysis (PCA). These kinematic parameters were sorted into separate PCA components and the obtained data matrix was rotated using the Varimax procedure with Kaiser normalization. As a result, regression scores for each PCA component were calculated per spermatozoon. On the basis of these regression scores, a two-step cluster analysis was run (log likelihood distance and Schwarz’s Bayesian criterion). This analysis identified up to three motile sperm subpopulations, and the percentages of spermatozoa belonging to each subpopulation (SP1, SP2, or SP3) were calculated. Again, a linear mixed model followed by post-hoc Sidak test was run to determine the effects of treatment (presence/absence of seminal plasma), storage time (48 and 72 h), and incubation (in vitro capacitation and progesterone-induced acrosomal exocytosis) on the percentages of SP1, SP2, and SP3 spermatozoa.

The level of significance was set at *p* ≤ 0.05 for all analysis, and data are shown as mean ± standard error of the mean (SEM).

## 5. Conclusions

Our results indicate that the presence of seminal plasma during liquid storage of pig semen at 17 °C modulated the sperm ability to elicit in vitro capacitation and trigger the acrosomal exocytosis induced by progesterone. In addition, this previous contact for 72 h led to decreased tyrosine phosphorylation of GSK3α/β, which appeared to be related to reduced mitochondrial membrane potential and ability to undergo acrosomal exocytosis. However, because the effects of the presence of seminal plasma during liquid storage 17 °C were less consistent in other sperm parameters, especially in the case of intracellular Ca^2+^ levels, further studies looking into in vitro fertilization (IVF) and artificial insemination outcomes are necessary to address whether it may be a beneficial practice for pig breeding.

## Figures and Tables

**Table 1:** Principal component analyses (PCA) based on individual kinematic parameters of all sperm cells evaluated by computer-assisted sperm analysis (CASA) system. As a result, two PCA components were obtained.

Principal Component	Variance	Parameter	a_ij_^2^
Component 1	58.04%	LIN	0.94
		WOB	0.81
		STR	0.79
		VSL	0.62
		VAP	0.48
		BCF	0.44
		MAD	0.28
Component 2	22.16%	ALH	0.88
		VCL	0.78
		DANCE	0.93
Total	80.20%		

The loading factor (a_ij_^2^) represents the highest association between a given sperm kinematic parameter and the corresponding principal component. LIN, linearity; WOB, wobble; STR, straightness; VSL, straight-line velocity; VAP, average path velocity; BCF, beat-cross frequency; MAD, mean angular displacement; ALH, amplitude of lateral head displacement; VCL, curvilinear velocity; DANCE, VCL × ALH.

**Table 2:** Descriptive statistics (mean ± SEM for seven experiments) of the three motile sperm subpopulations identified in this study.

	SP1	SP2	SP3
N	10,254	7458	3078
VCL (µm/s)	63.27 ± 0.27	92.87 ± 0.41	4.52 ± 0.19
VSL (µm/s)	49.91 ± 0.25	42.84 ± 0.44	2.86 ± 0.05
VAP (µm/s)	56.27 ± 0.27	61.39 ± 0.43	2.33 ± 0.10
LIN (%)	75.60 ± 0.15	41.49 ± 0.31	3.54 ± 0.16
STR (%)	86.47 ± 0.12	60.92 ± 0.33	6.91 ± 0.32
WOB (%)	86.94 ± 0.10	62.78 ± 0.25	8.76 ± 0.37
ALH (µm)	1.90 ± 0.01	3.57 ± 0.01	0.26 ± 0.01
BCF (Hz)	8.08 ± 0.03	7.79 ± 0.04	0.56 ± 0.03
DANCE (µm^2^/s)	129.28 ± 0.79	357.99 ± 2.72	7.60 ± 0.35
MAD (°)	64.78 ± 0.39	107.85 ± 0.50	90.35 ± 0.73

VCL, curvilinear velocity; VSL, straight-line velocity; VAP, average path velocity; (VAP/VCL); LIN, linearity; STR, straightness; WOB, wobble; ALH, amplitude of lateral head displacement; BCF, beat-cross frequency; DANCE (VCL × ALH); MAD, mean angular displacement.

## Abbreviations

ALH	amplitude of lateral head displacement
BCF	beat-cross frequency
BSA	bovine serum albumin
CASA	computer-assisted sperm analysis
CM	capacitating medium
DANCE	forward movement of the head
EV	electronic volume
FSC	forward scatter
GSK3a	glycogen synthase kinase-3 isoform α
GSK3ß	glycogen synthase kinase-3 isoform β
ISAS	integrated sperm analysis system
LIN	linearity
M540	merocyanine-540
MAD	mean angular displacement
PCA	principal component analysis
PI	propidium iodide
PMOT	progressive motility
SEM	standard error of the mean
SP	motile subpopulation
SRF	sperm-rich fraction
SSC	side scatter
STR	straightness
TMOT	total motility
VAP	average path velocity
VCL	curvilinear velocity
VSL	straight linear velocity
WOB	wobble coefficient
